# Quack Chemists

**Published:** 1871-04

**Authors:** 


### QUACK CHEMISTS.

Prof. F. W. Clark, in *Old & New*, gives a deserved thrashing to this class of humbugs. We give the following extracts from his article:

"In many cases, these certificates were given without even an attempt at analysis having been made. For example, a friend of the writer was one day in the office of a noted quack chemist in one of the New England States, when a stranger entered with a new nostrum to be analyzed. The chemist, scarcely glancing at the substance, asked its proprietor what it contained, and received a list of the ingredients in reply. Then, depending solely upon the word of the stranger, (quacks are proverbially truthful), a *certificate of analysis* was made out, paid for, and the fellow went on his way rejoicing. The guilty chemist in this case has attained to some celebrity, and by the outer world is regarded as a high authority. This sort of thing happens every day; and many of the certificates printed upon the labels of proprietary medicines are of this character, and are not worth the paper upon which they were first written.

"These certificates, however, vary in form, the absolute lie being probably rare. A favorite style among those quack chemists who fondly imagine themselves possessed of consciences, runs somewhat as follows: This is to certify that I have *examined* Mr. Smith's, Brown's, Jones's, or Robinson's (as the case may be) elixir, panacea, spirit, oil, or balm of a thousand humbugs (as the case may be), and find in it nothing of a deleterious character. The whole is wound up with a glowing panegyric (the degree of enthusiasm being measured by the amount of the fee received) upon the wonderful properties and virtues of the nostrum in question. Now, the analyticial part of this is literally true in most cases. The substance is examined,—the chemist looks at it, smells of it, and, if he is very courageous, tastes of it; and nothing injurious, in fact nothing whatever, is found. But the certificate is intended to convey the idea of analysis, and therefore is to all intents and purposes a lie; for to the crime of deception is added the disgrace of cowardice and hypocrisy. The truth is used as a mask for the falseoood.

"Many certificates, however, are given, which merely state that the nostrum examined is free from lead, mercury, silver, iron, or other metallic ingredients. These substances being easily detected, there is nothing necessarily false in such a certificate; and the value of the latter then depends wholly upon the character of the chemist giving it. It is to be hoped that they are usually true. But, notwithstanding these exceptional cases, the great majority of quacks' chemical certificates are absolutely worthless.

"It is hard to overestimate the harm done by this scientific swindling. Many of the most widely advertised 'remedies' are pernicious, not to say poisonous; and oftentimes serious illness, sometimes death, results from their use.

"At present, thoroughly educated, reliable chemists are loth to undertake 'job work' of any kind, preferring rather to obtain permanent situations as professors, superintendents of chemical works, or managers of assay-offices. They dare not give false certificates, or such as would be available to the venders of patent medicines; nor will they even run the risk of being confounded professionally with their disreputable (half) brethren.

"But it is not only in the analysis of unanalyzable nostrums that chemical quackery is evident; the same lack of conscience is in other kinds of work. The mining company whose mines are deficient in metal wish a better certificate than truth will allow, and straightway the quack chemist finds for them as much of the precious material as they desire in their ores. The most worthless minerals are found to be rich in everything, and a hundred per cent. of gold from pure quartz is quoted on the prospectus of the mine.

"But perhaps the most glaring example of scentific criminality may be found in the recommendation given by some chemists for dangerous and explosive napthas and petroleums. Here is a case in point. A dealer carried a sample of patent oil or burning-fluid to a well-known Massachusetts chemist, desiring a good recommendation, whereby he might secure better sales. The fluid was so inflammable that its vapor would ignite from a lamp placed ten feet from the bottle; and yet the chemist recommended it as an excellent article, much less explosive than other similar products which he had examined. Possibly the latter part of the assertion may have been true; but the bare recommendation of so dangerous an article was criminal."

---

THE REVIEW, of this city, has suspended—sorry.

